# Prognostic nutritional index and albuminuria in adults aged 20 years and above: a cross-sectional analysis in the United States

**DOI:** 10.3389/fnut.2024.1462789

**Published:** 2024-11-12

**Authors:** Zhimeng Jiang, Xingyu Zhu, Huixin Jiang, Donglin Zhao, Feifei Su

**Affiliations:** ^1^Graduate School of Hebei North University, Zhangjiakou, China; ^2^Department of Gastroenterology, Air Force Medical Center, Chinese People’s Liberation Army, Beijing, China; ^3^Department of Cardiovascular Medicine, Air Force Medical Center, Chinese People’s Liberation Army, Beijing, China; ^4^Haiyuan College of Kunming Medical University, Kunming, China

**Keywords:** albuminuria, cross-sectional study, inflammation, kidney injury, nutrition, prognostic nutritional index

## Abstract

**Background and objective:**

Albuminuria is an important early marker of kidney damage and progression of chronic kidney disease and is also linked to several chronic systemic diseases. The Prognostic Nutritional Index (PNI) is widely used in the assessment of multiple diseases. However, research dealing with the relationship between PNI and albuminuria remains scarce. This research project aims to examine this association.

**Methods and materials:**

The present study employed data from the National Health and Nutrition Examination Survey (NHANES) between 2017 and 2020, including 7,737 adult participants who met the study criteria. PNI was analyzed as a quartile-categorized variable. Multivariable regression models and smoothing curve fitting were adopted to examine the relationship between PNI and albuminuria. In order to ascertain the stability of the association across different populations, subgroup analyses were performed.

**Results:**

The study found a statistically significant inverse relationship between higher PNI levels and the prevalence of albuminuria. The fully adjusted model indicates that a one-unit increase in PNI is associated with a 4% reduced odds of albuminuria prevalence [0.96 (0.93, 0.98)]. Quartile analysis showed a stable inverse relationship, with the highest PNI quartile having the significantly lower odds of albuminuria prevalence [0.76 (0.62, 0.94), p for trend = 0.0004]. Smooth curve fitting and two-piecewise linear regression models indicated a nonlinear relationship between PNI and albuminuria, with a turning point at 42. Subgroup analysis confirmed the reliability of the inverse relationship between PNI and albuminuria across all groups.

**Conclusion:**

The findings of this study indicated that higher PNI levels are significantly inversely related to the odds prevalence of albuminuria. PNI could serve as an important predictor for the occurrence of albuminuria. Further prospective studies are needed to validate this association.

## Introduction

Albuminuria refers to the presence of abnormal amounts of albumin in the urine, typically expressed as an albumin-to-creatinine ratio (ACR) exceeding 30 mg/g (1). It acts as an early indicator of kidney damage and a significant predictor of the progression of chronic kidney disease (CKD) (2). Persistent high levels of albuminuria often indicate further deterioration of kidney function (3, 4). Albuminuria is also a complication of systemic chronic diseases such as diabetes and hypertension (5). Recent research indicates that persistent albuminuria is significantly correlated with adverse cardiovascular consequences, thereby establishing it not merely as a marker of renal health but also as an important indicator of systemic well-being. The prevalence of microalbuminuria figures between 5 and 19% in the general population, with rates as high as 23% observed in hypertensive patients and 40% in diabetic patients (6). The close association between albuminuria and CKD highlights its significance as a public health issue, emphasizing the importance of early clinical screening and intervention, especially in at-risk populations (7, 8).

.The Prognostic Nutritional Index (PNI) is a calculated value based on two variables: serum albumin levels and lymphocyte counts, and it evaluates an individual’s nutritional, immune, and inflammatory status. Initially, PNI was used to assess the prognosis of patients with gastrointestinal malignancies (9). Increasing evidence suggests that PNI is widely used in the assessment of various diseases, including mortality in coronary artery disease (10), survival rates in patients undergoing continuous renal replacement therapy (11), prognosis in cervical cancer (12), migraine (13), diabetic nephropathy in type 2 diabetes (14), and cognitive function in the elderly (15). Consequently, the potential clinical applications of PNI have attracted considerable interest from the medical community. The growing body of evidence lends support to the view that PNI has the potential to serve as a valuable biomarker in clinical practice.

Prior research has demonstrated a correlation between declining kidney function and the presence of systemic inflammation and nutritional status. A cohort study found that higher levels of some inflammatory markers (16–18) are associated with CKD. Notably, polymorphisms in proinflammatory cytokine genes have been associated with albuminuria in the Japanese general population, as evidenced by the Takahata study (19). This study suggests that genetic variations influencing inflammatory responses may contribute to the development of albuminuria. Similarly, the Framingham Offspring cohort study demonstrated a significant relationship between inflammation, kidney function, and albuminuria, reinforcing the notion that inflammatory processes play a crucial role in kidney health (20). Furthermore, recent findings indicate that podocyte-specific activation of the NOD-like receptor family, pyrin domain containing 3 inflammasome is a key contributor to diabetic kidney disease, highlighting the role of inflammation at the cellular level in the progression of renal complications (21). Malnutrition is a prevalent issue among individuals with renal insufficiency, and there is a significant association between malnutrition and elevated levels of inflammatory markers in these patients (22, 23). A correlation has been identified between micronutrient deficiencies and an increase in albuminuria. One such deficiency, vitamin D, has been demonstrated to promote albuminuria via its influence on inflammatory responses and immune functions (24, 25). Furthermore, evidence from studies indicates that administration of these micronutrients can mitigate kidney damage and improve albuminuria levels (26). However, there are few studies investigating the relationship between PNI and albuminuria. This study endeavors to investigate the relationship between PNI and albuminuria through the analysis of data derived from the National Health and Nutrition Examination Survey (NHANES). By addressing this gap in the literature, we hope to contribute valuable insights into the role of nutritional status in kidney health, thereby informing clinical interventions aimed at mitigating CKD risks.

## Methods and materials

### Study population

NHANES is a national cross-sectional survey using a multi-stage probability sampling design to provide representative data on the health and nutritional status of the non-institutionalized US population. The NHANES study was approved by the Ethics Review Board of the National Center for Health Statistics (NCHS), and all participants provided written informed consent prior to enrollment. Our analysis focused on adults aged 20 years and older, utilizing data collected during the NHANES cycles from 2017 to 2020.

**Figure 1:**
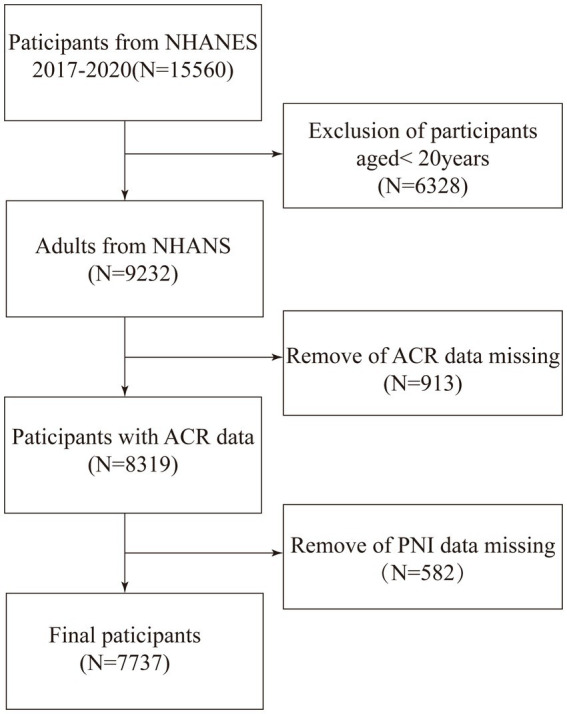
Flow diagram indicating study population.

Initially, a total of 15,560 eligible participants were recruited for the study. However, we excluded participants who were under 20 years of age (6,328 individuals), those with incomplete ACR data (913 individuals), and those with incomplete PNI data (582 individuals). As a result, 7,737 participants met the final study criteria (Figure 1).

### Exposure variables and outcome definition

In this study, the exposure variable was PNI, which was calculated using the following formula: PNI = 10 × albumin (g/dL) + 0.005 × absolute lymphocyte count (10^3^cells/μL). PNI was analyzed as a quartile-categorized variable. Albumin concentration was measured using bromocresol purple dye, and lymphocyte counts were obtained through the use of a Beckman Coulter DxH 800 analyzer.

Albuminuria was defined based on ACR, with a threshold of >30 mg/g, consistent with previous studies (27). Urinary albumin was determined via solid-phase fluorescence immunoassay, while urinary creatinine levels were quantified via modified Jaffe kinetic analysis.

### Covariates

In order to gain a deeper understanding of the relationship between PNI and albuminuria, we conducted a thorough review of existing cross-sectional studies and considered a range of potential confounding factors (26, 28, 29). These included demographic variables (age, gender, race, poverty income ratio, education level, and marital status), lifestyle factors (drinking status, smoking status, and moderate work activity status), and clinical measurements (white blood cell count, low-density lipoprotein cholesterol, total cholesterol, platelet count, high-density lipoprotein cholesterol, waist circumference, triglycerides, Body Mass Index (BMI), blood urea nitrogen, serum creatinine, uric acid, glomerular filtration rate, systolic blood pressure, diastolic blood pressure). Additionally, the presence of comorbid conditions such as diabetes, hypertension, hyperlipidemia, and kidney stones was recorded. All data generated by NHANES are freely accessible to the public via the CDC website.^1^

### Statistical analysis

In light of the intrinsic complexity of the NHANES sampling design, we employed the use of appropriate sampling weights. A weighted ANOVA was employed to assess between-group differences based on PNI quartiles for continuous variables, while a weighted chi-square test was applied for categorical variables. The objective was to ascertain whether there was a correlation between PNI and albuminuria by utilizing multivariable regression models. In Model 1, no covariates were included in the adjustments. Model 2 included adjustments for the covariates of age, gender, and race. In Model 3, all the covariates previously mentioned were included as adjustment variables including demographic variables, lifestyle factors, and clinical measurements. Sensitivity analysis was performed using PNI as a quartile-categorized variable. Smooth curve fitting was employed to evaluate the non-linear correlation between PNI and urinary protein. In the event of a nonlinear relationship being identified, a two-piecewise linear regression model was adopted for comparison with the single-line model (non-segmented). This involved a log-likelihood ratio test to assess the performance of the two models. The threshold effect size was calculated and tested for significance. Finally, subgroup analysis was carried out with the aim of idenfitying the stability of the relationship between PNI and albuminuria across different populations, with stratification by gender, age, race, BMI, diabetes, and hypertension. A *p*-value <0.05 was deemed to be statistically significant. All analyses were conducted utilizing R version 4.1.3 and Empower software.

## Results

### Baseline characteristics

Table 1 presented the weighted distribution of all clinical characteristics of the included participants, stratified by PNI quartiles. A total of 7,737 adult participants were included, with a 48.27 ± 17.22-year-old average age, 48.34% male and 51.66% female. The prevalence of hypertension and diabetes mellitus was significantly higher in the lower percentile PNI group and decreased with increasing PNI levels. Furthermore, age demonstrated a decreasing trend in PNI quartiles (*p* < 0.0001). No significant differences in BMI, diastolic blood pressure, and triglycerides were observed at varying PNI levels. The PNI quartile ranges were 21–38, 38–41, 41–43, and 43–54. The overall prevalence of albuminuria was 10.23%; the prevalence in quartiles 1, 2, 3, and 4 was 14.11, 11.06, 8.8, and 8.1%, respectively.

**Table 1:** Baseline characteristics of the study population (Location: United States, Years: 2017–2020).

Quartiles of prognostic nutritional index					
Characteristic	Q1 (21–38)	Q2 (38–41)	Q3 (41–43)	Q4 (43–54)	*p-value*
Age(years)	51.33 ± 16.97	51.67 ± 17.13	48.94 ± 16.73	42.78 ± 16.54	<0.0001
Ratio of family income to poverty	2.87 ± 1.62	3.08 ± 1.60	3.11 ± 1.53	3.29 ± 1.57	<0.0001
White blood cell count (10^3^cells/μL)	7.59 ± 2.36	7.27 ± 2.91	7.15 ± 7.17	7.33 ± 2.04	0.0195
Platelet count (10^3^cells/μL)	260.94 ± 72.36	246.41 ± 63.97	242.04 ± 57.39	241.16 ± 57.90	<0.0001
Uric acid (mg/dL)	5.20 ± 1.49	5.25 ± 1.50	5.34 ± 1.35	5.52 ± 1.40	<0.0001
Cholesterol (mg/dL)	182.46 ± 41.09	187.71 ± 40.63	188.91 ± 40.29	189.33 ± 40.91	<0.0001
Triglycerides (mg/dL)	140.41 ± 113.74	137.73 ± 89.51	140.18 ± 97.22	141.01 ± 106.53	0.7589
HDL-Cholesterol (mg/dL)	53.06 ± 15.35	54.15 ± 16.52	53.79 ± 15.67	53.90 ± 15.65	0.2160
LDL-Cholesterol (mg/dL)	104.38 ± 36.08	109.06 ± 36.32	110.18 ± 35.95	110.36 ± 35.71	<0.0001
Serum Creatinine (mg/dL)	14.75 ± 7.05	15.13 ± 5.55	15.13 ± 4.79	14.72 ± 4.71	0.0168
Blood Urea Nitrogen (mg/dL)	0.87 ± 0.50	0.89 ± 0.55	0.87 ± 0.20	0.88 ± 0.19	0.3846
Systolic Pressure (mmHg)	122.57 ± 19.61	122.41 ± 17.60	121.65 ± 16.56	121.40 ± 15.74	0.0973
Diastolic Pressure (mmHg)	74.66 ± 11.76	74.39 ± 10.72	74.32 ± 10.28	73.97 ± 10.37	0.2497
Waist circumference (cm)	88.41 ± 22.81	87.29 ± 21.64	88.82 ± 22.92	87.59 ± 22.96	0.1228
Glomerular filtration rate (ml/min)	95.42 ± 61.04	87.94 ± 52.83	94.60 ± 57.78	99.36 ± 57.67	<0.0001
Body mass index (kg/m^2^)	26.17 ± 7.93	25.90 ± 7.60	26.45 ± 7.99	26.14 ± 8.18	0.1964
Gender (%)					<0.0001
Male	30.20	41.85	50.71	64.16	
Female	69.80	58.15	49.29	35.84	
Race (%)					<0.0001
Mexican American	9.87	7.47	8.54	8.61	
Other Hispanic	8.25	6.98	7.93	7.76	
Non-Hispanic White	55.62	64.75	63.94	66.62	
Non-Hispanic Black	17.43	12.00	8.97	6.43	
Other race	8.83	8.79	10.62	10.58	
Education level (%)					<0.0001
Less than high school	13.62	10.83	11.46	8.73	
High school	29.30	28.44	24.00	26.27	
More than high school	57.07	60.72	64.54	65.00	
Marriage (%)					0.1988
Yes	61.46	61.36	64.34	62.30	
No	38.54	38.64	35.66	37.70	
Smoking (%)					0.0054
Yes	43.89	39.15	44.05	43.53	
No	56.11	60.85	55.95	56.47	
Drink (%)					0.1098
Yes	12.67	12.22	11.55	13.96	
No	87.33	87.78	88.45	86.04	
Moderate work activity (%)					0.0001
Yes	44.60	48.28	49.88	51.79	
No	55.40	51.72	50.12	48.21	
High blood pressure (%)					<0.0001
Yes	40.26	33.51	33.21	25.58	
No	59.74	66.49	66.79	74.42	
Diabetes (%)					<0.0001
Yes	17.61	12.78	10.72	6.79	
No	82.39	87.22	89.28	93.21	
Hypertriglyceridemia (%)					<0.0001
Yes	33.59	37.49	37.63	30.61	
No	66.41	62.51	62.37	69.39	
Kidney stone (%)					0.1218
Yes	11.08	10.35	10.71	8.96	
No	88.92	89.65	89.29	91.04	
Albuminuria (%)					<0.0001
Yes	14.11	11.06	8.80	8.10	
No	85.89	88.94	91.20	91.90	

Mean ± SD for continuous variables: the p value was calculated by the weighted linear regression model; (%) for categorical variables: the P value was calculated by the weighted chi-square test. HDL-Cholesterol, high-density lipoprotein cholesterol; LDL-Cholesterol, low-density lipoprotein cholesterol.

### Association between PNI and albuminuria

**Figure 2:**
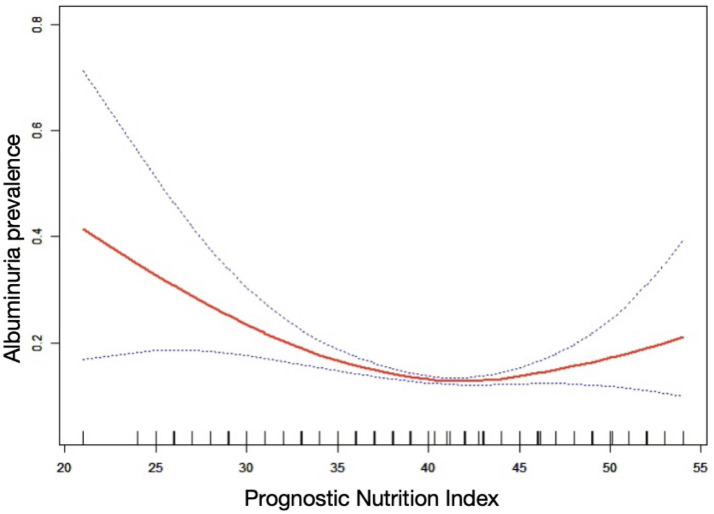
The nonlinear associations between PNI and albuminuria.

Table 2 demonstrated a negative correlation between PNI and the probability of albuminuria. In the unadjusted model [0.92 (0.90, 0.94)], the demographic-adjusted model [0.93 (0.91, 0.95)], and the fully adjusted model [0.96 (0.93, 0.98)], a negative correlation between PNI and albuminuria was observed. In the completely adjusted Model 3, a 4% decrease in the odds of albuminuria prevalence was observed with each unit increase in PNI, although the effect was slightly diminished, a significant negative correlation was still observed between PNI and albuminuria. When PNI was categorized into quartiles, a stable negative association was observed, which was statistically significant. The evidence provided thus far lends further support to the robustness of this correlation. A comparison of the lowest and highest PNI quartiles revealed a 24% lower prevalence of albuminuria in the latter [0.76 (0.62, 0.94), p for trend = 0.0004]. Although the data for the Q4 group demonstrated a robust association in both Models 1 and 2 (*p* < 0.0001), the observed association exhibited a slight reduction in strength but remained statistically significant following the adjustment for additional confounding variables in Model 3 (*p* = 0.0004). Nonlinear relationships between PNI and albuminuria were demonstrated by the smooth curve fitting analysis (Figure 2). The two-piecewise linear regression model showed a turning point at 42, with a log-likelihood ratio < 0.001 (Table 3).

**Table 2:** Associations between prognostic nutritional index and albuminuria (Location: United States, Years: 2017–2020).

Characteristic	Model 1 OR (95%CI)	Model 2 OR (95%CI)	Model 3 OR (95%CI)
PNI	0.92 (0.90, 0.94)	0.93 (0.91, 0.95)	0.96 (0.93, 0.98)
Categories			
Q1	1.0	1.0	1.0
Q2	0.68 (0.57, 0.81)	0.67 (0.56, 0.80)	0.78 (0.65, 0.95)
Q3	0.52 (0.43, 0.62)	0.55 (0.45, 0.66)	0.63 (0.51, 0.77)
Q4	0.52 (0.44, 0.63)	0.65 (0.53, 0.79)	0.76 (0.62, 0.94)
PNI group trend	<0.0001	<0.0001	0.0004

Model 1: no covariates were adjusted. Model 2: age, gender, and race were adjusted. Model 3: gender, age, race, family income-to-poverty ratio, education level, physical activity status, marital status, drink status, smoking status, systolic blood pressure, diastolic blood pressure, serum creatinine, serum urea nitrogen, white blood cell count, Platelet count, uric acid, cholesterol, triglycerides, HDL-Cholesterol, LDL-Cholesterol, glomerular filtration rate, body mass index, waist circumference, and history of diabetes, hypertension, hyperlipidemia, and kidney stones were adjusted. PNI, prognostic nutritional index; HDL-Cholesterol, high-density lipoprotein cholesterol; LDL-Cholesterol, low-density lipoprotein cholesterol.

**Table 3:** Threshold effect analysis of prognostic nutritional index on albuminuria using a two-piecewise linear regression model (Location: United States, Years: 2017–2020).

Model	OR (95%CI)	*p*- value
Inflection point (K)	42	
Fitting by two-piecewise linear model		
PNI ≤ 42	0.94 (0.91, 0.97)	<0.0001
PNI > 42	1.10 (1.03, 1.16)	0.0018
Log-likelihood ratio		<0.001

Model: gender, age, race, family income-to-poverty ratio, education level, physical activity status, marital status, drink status, smoking status, systolic blood pressure, diastolic blood pressure, serum creatinine, serum urea nitrogen, white blood cell count, Platelet count, uric acid, cholesterol, triglycerides, HDL-Cholesterol, LDL-Cholesterol, glomerular filtration rate, body mass index, waist circumference, and history of diabetes, hypertension, hyperlipidemia, and kidney stones were adjusted. PNI, prognostic nutritional index; HDL-Cholesterol, high-density lipoprotein cholesterol; LDL-Cholesterol, low-density lipoprotein cholesterol.

### Subgroup analysis

Subgroup analyses showed that PNI was inversely related to albuminuria in all subgroups. Although some differences in the association between PNI and albuminuria were observed between the various subgroups, the interaction analyses demonstrated that these differences did not reach statistical significance (*p*-value >0.05 for all interactions). This suggests that the correlation between PNI and albuminuria was largely consistent, although the impact may have been more pronounced in certain subgroups. For instance, the correlation was less pronounced in female subjects in the sex-stratified analysis and did not attain statistical significance (*p* = 0.1691). Furthermore, the correlation between PNI and albuminuria was also not statistically significant in the 20–40 age group, individuals with normal weight, overweight individuals, individuals with diabetes mellitus, and certain racial categories (non-Hispanic whites and other races; Table 4).

**Table 4:** Subgroup analysis of the association between prognostic nutritional index and albuminuria (Location: United States, Years: 2017–2020).

Characteristic	Model OR (95%CI)	*p*- value	*p* for interaction
Stratified by age (years)			0.8195
20–40	0.97 (0.93, 1.02)	0.2436	
40–60	0.96 (0.92, 0.99)	0.0171	
60–80	0.96 (0.93, 0.99)	0.0185	
Stratified by gender			0.5513
Male	0.97 (0.94, 1.00)	0.0346	
Female	0.98 (0.95, 1.01)	0.1691	
Stratified by race			0.1355
Mexican American	0.94 (0.89, 1.00)	0.0338	
Other Hispanic	0.91 (0.85, 0.98)	0.0109	
Non-Hispanic White	0.99 (0.95, 1.03)	0.6246	
Non-Hispanic Black	0.95 (0.91, 0.99)	0.0153	
Other race	0.99 (0.94, 1.05)	0.8147	
Stratified by BMI			0.8127
Normal weight	0.97 (0.94, 1.00)	0.0957	
Overweight	0.96 (0.93, 1.00)	0.0521	
Obese	0.96 (0.92, 1.00)	0.0311	
Stratified by diabetes			0.9799
Yes	0.97 (0.93, 1.00)	0.0712	
No	0.97 (0.94, 0.99)	0.0084	
Stratified by high blood pressure			0.8844
Yes	0.96 (0.94, 0.99)	0.0128	
No	0.97 (0.94, 1.00)	0.0419	

Model: gender, age, race, family income-to-poverty ratio, education level, physical activity status, marital status, drink status, smoking status, systolic blood pressure, diastolic blood pressure, serum creatinine, serum urea nitrogen, white blood cell count, Platelet count, uric acid, cholesterol, triglycerides, HDL-Cholesterol, LDL-Cholesterol, glomerular filtration rate, body mass index, waist circumference, and history of diabetes, hypertension, hyperlipidemia, and kidney stones were adjusted. PNI, prognostic nutritional index; HDL-Cholesterol, high-density lipoprotein cholesterol; LDL-Cholesterol, low-density lipoprotein cholesterol.

## Discussion

This study, which included 7,737 adult participants from the NHANES, aimed to assess the association between PNI and albuminuria. We observed that higher PNI levels were related to a lower odds prevalence of albuminuria. This negative association was still found to be stable in the completely adjusted model. Non-linear association between PNI and albuminuria suggested by smooth curve fitting, with a turning point at 42. No significant interaction effects were found in different subgroups, indicating that this inverse relationship was consistent across different populations. Our findings suggest that PNI can predict the occurrence of albuminuria. For individuals with low PNI levels, early management and intervention in nutrition and inflammation may reduce the incidence of albuminuria and slow the progression of kidney damage.

PNI is a composite index that is simple and easy to obtain, with broad clinical applications. PNI levels are associated with the risk and progression of kidney damage, according to existing studies. A meta-analysis conducted by Chen et al. revealed that PNI is a valuable predictive marker for acute kidney injury (AKI) in critically ill patients, with higher sensitivity in CKD patients compared to non-CKD patients (30). A retrospective cohort study by Zhang et al. involving 14,349 participants demonstrated that PNI serves as an independently prognostic factor for all-cause mortality in diabetic nephropathy patients. The study found that increased PNI levels were associated with a reduced risk of mortality [0.64, (0.459,0.892), *p* = 0.01]. PNI can also be employed as an efficacious indicator for the prediction of AKI resulting from the administration of contrast media in patients diagnosed with coronary artery disease (31, 32). Another retrospective study including 3,360 CKD patients, showed that elevated PNI were inversely correlated with mortality risk, with mortality decreasing in an “L” shape as PNI levels increased (33). These studies suggest that PNI has potential clinical value in predicting kidney damage.

PNI is composed of two critical factors: albumin and lymphocytes, which assess the body’s inflammation, nutrition, and immune status. The mechanisms underlying the relationship between PNI and albuminuria remain unclear. Existing studies suggest that inflammation and nutritional status are closely related to kidney function, which may explain the potential mechanisms between PNI and albuminuria. A 15-year follow-up cohort study including 4,926 participants demonstrated that *in vitro* inflammatory markers, including C-reactive protein, interleukin-6(IL-6), tumor necrosis factor-*α* (TNF-α), and white blood cell, were positively associated with a heightened risk of CKD (17). Furthermore, animal studies have indicated that inflammation is associated with declining kidney function. In IL-6 transgenic mice, overexpression of IL-6 promotes inflammation, mediates tubulointerstitial lesions, potentially affects glomerular damage, and increases oxidative stress and apoptosis, leading to progressive kidney injury and multiple myeloma kidney disease development (34). Another study in an inflammatory rat model showed that elevated TNF and interleukin-1 (IL-1) levels increased the severity of glomerular damage in nephritis (35). Pro-inflammatory cytokines accelerate protein degradation while inhibiting protein synthesis, leading to an imbalance that increases protein consumption and malnutrition (36). Serum albumin, another component of PNI, reflects an individual’s nutritional status. When glomerular filtration function is impaired, large amounts of protein, especially albumin, are lost in the urine, leading to decreased serum albumin levels. Continuous protein loss can cause malnutrition, decreased immune function, increased risk of infections, and other complications, further damaging kidney function (22). Malnutrition not only reduces protein synthesis but also affects kidney repair and regeneration (37). Serum albumin also has antioxidant and anti-inflammatory properties (38). A reduction in serum albumin levels is associated with an increase in inflammatory markers (39), with inflammatory cytokines including IL-1, IL-6, and TNF-*α* linked to decreased serum albumin levels (40). Normal physiological levels of albumin can inhibit the expression of vascular cell adhesion molecules induced by TNF-α, thereby reducing inflammation (41). Low albumin levels in diabetic patients may also accelerate oxidative stress and inflammatory responses, worsening kidney function (42, 43). A retrospective study by Zhang et al. indicated that diabetic nephropathy patients with low serum albumin levels were more likely to progress to end-stage renal disease (39). Similarly, albuminuria is an important predictor of poor prognosis in CKD patients (39, 44, 45). In summary, inflammation can cause malnutrition, and malnutrition can lead to decreased immune function, exacerbating inflammation in a vicious cycle, which in turn causes renal insufficiency and albuminuria.

A significant negative correlation was observed between PNI and albuminuria. Nevertheless, subgroup analyses demonstrated discrepancies in the magnitude and statistical significance of the association across gender, age, and ethnic groups. To illustrate, a significant correlation was identified between reduced PNI and elevated albuminuria in male subjects, whereas this association was not statistically significant in female subjects. This may be attributable to physiological differences between men and women and their disparate nutritional intake and health behaviors. Furthermore, no significant association was observed within the age group 20–40 years, which may be attributed to the fact that this age group typically exhibits superior health status, thereby attenuating the impact of PNI on albuminuria. Additionally, no significant associations were identified for individuals with normal weight, overweight status, diabetes mellitus, or among certain racial groups (non-Hispanic whites, other race). Further investigation into the underlying biological and socioeconomic factors may be necessary to elucidate these observations.

The study’s notable strengths include a large and representative sample size and the adjustment of potential confounders to provide accurate and reliable results. Nevertheless, a causal relationship between PNI and albuminuria cannot be established due to the cross-sectional design of the study. Secondly, despite the inclusion of numerous confounders, there are still some significant influences that remain uncontrolled for, such as medications and diseases that affect albumin levels. These unincluded confounders may have an impact on the association between PNI and albuminuria. Furthermore, PNI, as a static indicator, reflects the current nutritional status of an individual, but it does not capture its changes over time. Given that PNI can fluctuate in response to an individual’s health status and lifestyle habits, data obtained at a single point in time may not fully reflect the long-term nutritional status and its impact on health.

## Conclusion

Our study indicated that higher PNI levels are associated with reduced albuminuria excretion. Our study lends support to the hypothesis that nutritional status may play a role in the progression of kidney injury. However, to gain a more comprehensive understanding of this relationship and to incorporate additional influencing factors, it would be beneficial to conduct prospective studies and explore the causal mechanisms of this association. Such studies should aim to identify the role of nutritional status and inflammatory markers in influencing PNI and albuminuria. Additionally, it would be beneficial to investigate the potential clinical applications of PNI in managing nutrition and inflammation in patients with CKD and other related conditions. By incorporating a diverse range of populations and considering various confounding factors, future research can provide more generalized findings. Furthermore, interventions aimed at improving nutritional status and reducing inflammation should be evaluated for their effectiveness in lowering albuminuria levels, potentially leading to new strategies for preventing CKD progression.

## Data Availability

Publicly available datasets were analyzed in this study. This data can be found at: www.cdc.gov/nchs/nhanes/.

## Footnotes

^1^https://www.cdc.gov/nchs/nhanes/
